# Which surgical approach is more favorable for pheochromocytoma of different sizes (< 6 cm vs. ≥ 6 cm)? A single retrospective center experience

**DOI:** 10.1186/s12957-023-03164-w

**Published:** 2023-09-11

**Authors:** Shun Wan, Kunpeng Li, Chenyang Wang, Siyu Chen, Huabin Wang, Yao Luo, Xiaoran Li, Li Yang

**Affiliations:** 1 Department of Urology, Lanzhou University Second Hospital, Lanzhou, 730000 China; 2 Gansu Province Clinical Research Center for Urology, Lanzhou, 730000 China

**Keywords:** Pheochromocytoma, Laparoscopic adrenalectomy, Lateral transperitoneal adrenalectomy, Posterior retroperitoneoscopic adrenalectomy, Different sizes

## Abstract

**Background:**

To compare the surgical effects of lateral transperitoneal approach (LTA) and posterior retroperitoneal approach (PRA) for pheochromocytoma of different sizes.

**Methods:**

Data on patients with pheochromocytoma from 2014 to 2023 were collected from our hospital. According to different surgical approaches and tumor size, all patients were divided into four groups: tumor size < 6 cm for LTA and PRA and tumor size ≥ 6 cm for LTA and PRA. We compared these two surgical methods for pheochromocytoma of different sizes.

**Results:**

A total of 118 patients with pheochromocytoma underwent successful laparoscopic surgery, including PRA group (*n* = 80) and LTA group (*n* = 38). In tumor size < 6 cm, the outcomes were no significant difference in LTA and PRA. In tumor size ≥ 6 cm, there was a significant difference in operation time (214.7 ± 18.9 vs. 154.3 ± 8.2, *P* = 0.007) and intraoperative blood loss (616.4 ± 181.3 vs. 201.4 ± 45.8, *P* = 0.037) between LTA and PRA.

**Conclusion:**

LTA and PRA were performed safely with similar operative outcomes in patients with pheochromocytoma size < 6 cm. While both LTA and PRA were executed with a commendable safety profile and comparable operative results in patients afflicted by pheochromocytomas < 6 cm, the PRA technique distinctly showcased advantages when addressing large-scale pheochromocytomas (≥ 6 cm). Notably, this manifested in reduced operative time, diminished intraoperative blood loss, decreased hospitalization expenses, and a paucity of procedural complications.

## Introduction

Pheochromocytoma is a catecholamine-secreting tumor arising from the chromaffin tissue of the adrenal medulla. Because of its powerful sympathetic stimulation, it leads to clinical symptoms such as palpitations, tachycardia, hypertension, and dizziness in patients [1]. The estimated annual incidence of pheochromocytoma is 2–9.1 per 1 million adults [2]. Pheochromocytoma has clear surgical indications, and about 90% of pheochromocytomas are benign tumors, so the long-term postoperative outcome is good. However, due to the functional characteristics of pheochromocytoma, severe intraoperative blood pressure fluctuations are the main reason for its higher risk of surgery than other types of adrenal tumor surgery. Preoperative use of α-blockers is required to expand blood volume to reduce intraoperative blood pressure fluctuations fully [3]. Surgical excision, besides addressing the tumor, effectively ameliorates clinical symptoms [4]. Several studies have demonstrated superior laparoscopic adrenalectomy (LA) results compared to open adrenalectomy in treating pheochromocytoma [5, 6]. A decade-long follow-up of 118 pheochromocytoma patients post-LA revealed a mere 2.6% succumbing to metastatic pheochromocytoma and a paltry 0.8% encountering lymph node metastasis [7]. Echoing this, the study by Falhammar et al. unveiled that 70% of their pheochromocytoma cohort underwent laparoscopic interventions. Their median 8-year follow-up witnessed a 13% mortality rate stemming from metastatic pheochromocytoma [8]. Aggregating insights through a meta-analysis of 34 distinct investigations, the global recurrence incidence (with a median time span of 5 years) ensuing LA stood at 0.98 events per 100 person-years [9], with no appreciable divergence from open surgery outcomes.

LA has now emerged as a standardized treatment for pheochromocytoma [10]. While there has been contemplation surrounding the application of LA for pheochromocytomas of larger dimensions (≥ 6 cm), owing to potential precipitous hemodynamic perturbations, its advisability has been contested [11]. Notwithstanding, LA perseveres as a secure and efficacious option for larger pheochromocytomas, as corroborated by meta-analytic findings [10]. There are two surgical methods for laparoscopic resection of the pheochromocytoma: lateral transperitoneal approach (LTA) and posterior retroperitoneal approach (PRA). Currently, there is no standard and basis for the approach selection, and each endoscopic center selects according to its learning curve and habits [12]. While a handful of published studies have endeavored to draw a comparison between LTA and PRA for pheochromocytoma, their conclusions remain disparate [13]. Gockel et al. reported shorter operative time, lower peak intraoperative blood pressure frequency, and a better overview of the surgical field for LTA. They recommended LTA as the procedure of choice for pheochromocytoma [14]. A 2023 meta-analysis, however, did underscore the supremacy of LTA over PRA for larger pheochromocytomas (≥ 6 cm) [10]. Conversely, a comprehensive meta-analysis has illuminated PRA’s superior perioperative outcomes in the context of pheochromocytoma patients compared to LTA [15]. However, tumor size was not categorized in these studies.

The existing investigations involving LTA and PRA in the context of pheochromocytoma suffer from limited cohort sizes, diverse surgical operators, thereby bearing certain study limitations. Furthermore, there is a paucity of studies that have systematically contrasted perioperative outcomes after stratification based on tumor size in the context of pheochromocytoma. In this light, the present study retrospectively gathers data from 118 patients who underwent pheochromocytoma resection at the Lanzhou University Second Hospital. The overarching goal is to furnish an objective clinical basis for surgical approach selection and guide judicious clinical decision-making.

## Methods

A total of 118 patients who underwent laparoscopic resection of pheochromocytoma in the department of urology of our hospital from November 2014 to January 2023 were selected as the study subjects. The included patients were operated by same chief physician. All patients signed surgical consent. The inclusion criteria were as follows: benign pheochromocytoma confirmed by postoperative pathology according to the patient’s clinical symptoms, signs, laboratory tests, and imaging diagnosis, all were unilateral benign lesions, no history of abdominal surgery in the same quadrant of adrenal tumor location, and voluntarily signed a surgical informed consent form. The exclusion criteria were as follows: requiring other surgeries simultaneously or with persistent infection, malignant pheochromocytoma, postoperative pathological diagnosis of non-pheochromocytoma, transfer to open surgery, suspected adrenal cancer, and combined cardiac, hepatic, and renal dysfunction.

### Preoperative preparation

In the diagnostic trajectory of pheochromocytoma, a composite evaluation integrating urinary catecholamine assessment and contrast-enhanced CT scan imaging serves as the bedrock, with the ultimate diagnostic veracity confirmed through histopathological examination. In preoperative routine examination of blood electrolytes and adrenal-related hormones, patients with hypokalemia were given spironolactone to correct the electrolyte imbalance. All patients controlled for hypertension were given α-receptor blocker phenoxybenzamine (60 ~ 120 mg/day, divided 3 ~ 4 times orally) for sufficient blood size expansion. Calcium antagonists were used as appropriate to stabilize the blood pressure at about 130/80 mmHg. The above preoperative preparation time was 1 ~ 2 weeks, and preoperative-related complications were also treated (such as headache, tachycardia, hypertension).

### Surgical method

#### PRA

All patients were given general anesthesia and blood pressure control, indwelling catheter, without the routine indwelling gastric tube. The patients were placed in a healthy lateral decubitus position. After routine skin incision, 12-mm trocar was used 2 cm below the costal margin of the right posterior axillary line for puncture. A self-made balloon dilated the retroperitoneal operating space. Then, 5-mm and 12-mm trocar was used from 2 cm below the costal margin of the anterior axillary line and 2 cm above the middle axillary, iliac crest under the guidance of the index finger to establish a thick line fixation after pneumoperitoneum. The ultrasound knife incised the posterior renal fascia longitudinally, freed it along the posterior aspect of the kidney and upward from the psoas muscle to the upper pole of the kidney, freed the adrenal tumor, and clamped Ham-o-lok to the vessels connected to the surface of the tumor. If the blood vessels on the surface of the tumor bleed, the supplying blood vessels are gradually separated with an ultrasonic knife, then the blood vessels are ligated with Ham-o-lok, and finally, the whole tumor is wholly separated and resected. The whole tumor was completely freed and resected, placed in a self-made specimen bag, and removed from the body along the incision of the posterior axillary line. The operator carefully checked during the operation to ensure complete resection of the entire tumor and placement of a drainage tube after hemostasis.

#### LTA

All patients underwent general anesthesia, indwelling catheter without the routine indwelling gastric tube. Place the patient in the 70° healthy lateral position. Transverse incision of 1 cm of the skin above the left anterior superior iliac spine was performed to establish pneumoperitoneum with Veres’s needle puncture (15 mmHg). Insert 10-mm trocar and a laparoscope, respectively; place a 5-mm and 12-mm trocar under the costal margin of anterior axillary line and midclavicular line under monitoring; and if necessary, place a 5-mm trocar at the umbilicus of the midaxillary line. Ultrasonic scalpel incises the peritoneum and paracolic gutter, retracts the hepatic flexure/splenic flexure inferiorly, and pushes the liver/spleen upward. The perirenal fascia was cut with a super knife to expose the adrenal area. The adrenal glands were separated, and the blood vessels connected with Ham-o-lok were ligated. The tumor was freed entirely and removed through a self-made specimen bag at 12-mm trocar. The interactive examination was performed to ensure complete resection of the entire tumor and complete hemostasis. In PRA and LTA, the main principle is to remove as much perirenal tissue as possible to prevent malignant tumors. A drain is routinely placed before the procedure is completed.

### Observation indicators

The primary outcome of this study was operative time. Preoperative and postoperative blood pressure, intraoperative blood loss, intraoperative hemodynamic stability, postoperative pain, a conversion rate of open surgery, length of postoperative hospitalization, postoperative time of drainage tube removal, the incidence of complications, and hospitalization costs were followed. Operative time is calculated from the first incision to the last stitch. Blood loss was calculated as suction and gauze weight.

### Statistics

Statistical analysis was performed using IBM® SPSS version 25 (SPSS Inc., USA). Enumeration data are expressed as a percentage. The Kolmogorov–Smirnov test was used to determine whether continuous variables were normally distributed and expressed as mean ± standard deviation or median, and Student’s *t*-test or Mann–Whitney *U*-test was used for analysis. The Pearson chi-square test and Fisher exact test were used to comparing the categorical variables. For multivariate analysis, continuous variables such as age, BMI, and tumor size were dichotomized by the median value. When *P* < 0.05, there was a significant difference.

## Results

### Clinical characteristics

A total of 118 patients with pheochromocytoma underwent successful laparoscopic surgery, PRA group (*n* = 80), including tumor size < 6 cm (*n* = 52) and size ≥ 6 cm (*n* = 28) and LTA group (*n* = 38), including tumor size < 6 cm (*n* = 17) and size ≥ 6 cm (*n* = 21). Table 1 summarizes the baseline clinical characteristics of the enrolled patients. The LTA group was significantly more than the PRA group in the proportion of patients with BMI ≥ 24 kg/m^2^, the LTA group was significantly less than the PRA group in the proportion of patients with 18.5 kg/m^2^ ≤ BMI < 24 kg/m^2^, and there was no significant difference between the two groups in other clinical data.

**Table 1:** Characteristics of the participants

	LTA (*n* = 38)	PRA (*n* = 80)	*P*
Sex			
Male	12 (31.6%)	36 (45.0%)	0.16
Female	26 (68.4%)	44 (55.0%)	0.24
Age/years	49.0 ± 13.2	47.2 ± 13.1	0.86
BMI/kg/m^2^			
< 18.5	1 (2.6%)	5 (6.3%)	0.86
18.5 ≤ BMI < 24	10 (26.3%)	67 (83.8%)	0.01
≥ 24	27 (71.1%)	8 (10%)	0.01
Preoperative hypertension	24 (63.2%)	54 (67.5%)	0.81
Preoperative diabetes	5 (13.1%)	14 (17.5%)	0.82
History of abdominal surgery	7 (18.4%)	17 (21.3%)	0.91
Preoperative systolic blood pressure/mmHg	124.7 ± 21.2	131.3 ± 21.5	0.76
Preoperative diastolic blood pressure/mmHg	81.2 ± 13.6	83.2 ± 14.4	0.85
Tumor location			
Right	21 (55.3%)	54 (67.5%)	0.52
Left	17 (44.7%)	26 (32.5%)	0.41
Tumor size/cm	5.8 ± 2.4	4.7 ± 2.1	0.26

*LTA* indicates lateral transperitoneal adrenalectomy, *PRA* posterior retroperitoneoscopic adrenalectomy, *BMI* body mass index

### Outcome

In tumor size < 6 cm, there was no significant difference in LTA and PRA operation time (155.3 ± 35.6 vs. 153.5 ± 51.6, *P* = 0.896), intraoperative blood loss, tumor size, and intraoperative hemodynamics (Table 2). However, patients with higher BMI values were likelier to choose LTA (*P* < 0.05). In tumor size ≥ 6 cm, there was a significant difference in operation time (214.7 ± 18.9 vs. 154.3 ± 8.2, *P* = 0.007) and intraoperative blood loss (616.4 ± 181.3 vs. 201.4 ± 45.8, *P* = 0.037) between LTA and PRA groups, and the difference was statistically significant (*P* < 0.05) (Table 2). Similar to previous results, patients with higher BMI values were likelier to choose LTA (*P* < 0.05). In tumor size ≥ 6 cm, the incidence rate of postoperative complications and hospitalization costs in the PRA group was less than that in the LTA group, and the differences had statistical significance (*P* < 0.05). The main complications occurred were arrhythmia, gastrointestinal symptoms, respiratory symptoms, and adrenal crisis, higher costs when pneumonia and arrhythmia occur. However, there was no significant difference in a postoperative hospital stay and drainage tube removal time between the two groups (Table 3).

**Table 2:** Operative outcomes in different sizes (< 6 cm vs. ≥ 6 cm) group: LTA versus PRA

	Tumor size < 6 cm			Tumor size ≥ 6 cm		
	LTA (*n* = 17)	PRA (*n* = 52)	*P*	LTA (*n* = 21)	PRA (*n* = 28)	*P*
**Tumor size/cm**	3.7 ± 1.2	3.6 ± 1.2	0.865	7.6 ± 1.6	6.8 ± 1.8	0.143
**BMI/ kg/m**^**2**^						
**< 18.5**	1 (5.9%)	1 (1.9%)	0.912	0 (0%)	4 (14.3%)	0.912
**18.5 ≤ BMI < 24**	7 (41.2%)	43 (82.7%)	0.008	3 (14.3%)	24 (85.7%)	0.0012
**≥ 24**	9 (52.9%)	8 (15.4%)	0.018	18 (85.7%)	0 (0%)	< 0.001
**Conversions/n**	1 (5.9%)	0 (0%)	0.836	1 (4.8%)	3 (10.7%)	0.683
**Operative time/min**	155.3 ± 35.6	153.5 ± 51.6	0.896	214.7 ± 18.9a	154.3 ± 8.2a	0.007
**Blood loss/ml**	132.9 ± 29.1	139.6 ± 25.1	0.888	616.4 ± 181.3	201.4 ± 45.8	0.037
**Intraoperative highest SBP/mmHg**	188.8 ± 23.9	175.3 ± 17.3	0.218	175.6 ± 8.6	174.1 ± 15.1	0.801
**Intraoperative lowest SBP/mmHg**	83.8 ± 4.7	82.5 ± 4.3	0.327	73.8 ± 14.2	78.6 ± 10.3	0.768
**Intraoperative highest DBP/mmHg**	113.3 ± 7.6	105.1 ± 8.9	0.158	101.7 ± 9.3	105.0 ± 17.6	0.686
**Intraoperative lowest DBP/mmHg**	72.5 ± 17.7	55.0 ± 15.0	0.237	45.0 ± 5.0	50.0 ± 4.2	0.413
**Intraoperative highest heart rates**	128.3 ± 20.2	110.0 ± 9.4	0.253	123.8 ± 16.0	135.0 ± 18.3	0.574
**Intraoperative lowest heart rates**	49.6 ± 8.5	42.2 ± 7.3	0.141	42.5 ± 6.7	45.4 ± 8.2	0.342

*SBP* indicates systolic blood pressure, *DBP* diastolic blood pressure, *LTA* lateral transperitoneal adrenalectomy, *PRA* posterior retroperitoneoscopic adrenalectomy, *BMI* body mass index

**Table 3:** Comparison of postoperative conditions in different sizes (< 6 cm vs. ≥ 6 cm) group: LTA versus PRA

	Tumor size < 6 cm			Tumor size ≥ 6 cm		
	LTA (*n* = 17)	PRA (*n* = 52)	*P*	LTA (*n* = 21)	PRA (*n* = 28)	*P*
**Length of hospitalization/day**	6.8 ± 1.7	7.1 ± 2.2	0.544	8.9 ± 3.9	7.1 ± 2.3	0.051
**Time for removal of drainage tube/day**	4.1 ± 1.5	4.3 ± 2.0	0.801	5.8 ± 2.8	4.5 ± 2.0	0.081
**Complication rate**	4 (5.8%)	9 (13%)	0.569	9 (18.4%)	2 (4.1%)	0.003
**Hospitalization cost/USD**	3716 ± 986	3862 ± 864	0.593	4668 ± 1340	3807 ± 927	0.016

*LTA* indicates lateral transperitoneal adrenalectomy, *PRA* posterior retroperitoneoscopic adrenalectomy

### Univariate and multivariate analysis

In the realm of univariate and multivariate analysis, a cohort of 70 patients with operative durations exceeding 150 min was considered. The findings of the multivariate logistic regression analysis illuminated that attributes such as female gender [odds ratio (OR) = 0.29, 95% *CI* = 0.31–2.10; *P* = 0.02], selection of LTA [*OR* = 4.25, 95% *CI* = 1.39–12.98; *P* = 0.01], and blood loss surpassing 100 ml [*OR* = 1.01, 95% *CI* = 0.98–1.01; *P* < 0.01] were intricately intertwined with operative durations surpassing the 150 min threshold. The examination of secondary outcomes yielded no substantive disparities among the various groups (Table 4).

**Table 4:** Univariate and multivariate analysis of clinical factors associated with operative time ≥ 150 min

Characteristics	Univariate analysis			Multivariate analysis		
	**OR**	**95% ** ***CI***	***P***	**OR**	**95% ** ***CI***	***P***
**Sex (female)**	0.51	0.24–1.10	0.09	0.29	0.31–2.10	0.02
**Age (**≥ **46)**	0.93	0.44–1.96	0.84	1.24	0.48–3.24	0.66
**BMI (≥ 24)**	0.82	0.42–1.86	0.56	0.41	0.13–1.21	0.15
**History of abdominal surgery (yes)**	1.88	0.71–4.96	0.20	2.90	0.91–9.22	0.07
**Preoperative hypertension (yes)**	0.93	0.42–2.04	0.85	0.92	0.28–3.04	0.89
**Preoperative diabetes (yes)**	1.45	0.50–4.17	0.49	1.64	0.43–6.16	0.47
**Tumor location (left)**	1.72	0.79–3.76	0.18	1.73	0.65–4.58	0.27
**Tumor size (≥ 6)**	1.59	0.74–3.41	0.23	0.60	0.22–1.67	0.33
**Preoperative SBP (> 125)**	1.13	0.54–2.36	0.75	2.68	0.74–9.66	0.13
**Preoperative DBP (> 82)**	0.82	0.39–1.71	0.59	0.44	0.15–1.32	0.14
**Operative method (LTA)**	2.85	1.23–6.62	0.02	4.25	1.39–12.98	0.01
**Blood loss (> 100 ml)**	0.86	0.78–1.00	< 0.01	1.01	0.98–1.01	< 0.01

*SBP* indicates systolic blood pressure, *DBP* diastolic blood pressure, *LTA* lateral transperitoneal adrenalectomy, *PRA* posterior retroperitoneoscopic adrenalectomy, *BMI* body mass index

## Discussion

Pheochromocytoma is a common manifestation of paroxysmal or persistent hypertension caused by excessive catecholamine synthesis, secretion, and release into the blood by chromaffin cells in the adrenal medulla. Surgical treatment is the main treatment for pheochromocytoma, and early surgical resection of the tumor is the only way to clinical cure [16]. Laparoscopic pheochromocytoma resection has two procedures: PRA and LTA, and which procedure has greater advantages remains controversial. In a study involving 42 pediatric cases of pheochromocytoma, both PRA and LTA demonstrated safety and efficacy. Although results demonstrated the safety and efficacy of either surgical approach in pediatric cases, this investigation refrained from directly contrasting the two modalities, hindered in part by its limited case volume [17]. In the current study, encompassing a total of 118 pheochromocytoma patients, surgeons have been chief physicians for over 5 years. Our findings stand in concordance, affirming the safety and effectiveness of both LTA and PRA as minimally invasive treatments across the spectrum of small (< 6 cm) and large (≥ 6 cm) pheochromocytomas.

Cabalag et al., in their exploration involving 49 pheochromocytoma cases, unveiled the shorter relative operative times of PRA, an observation paralleled by diminished postoperative analgesic requirements and abbreviated hospital stays [18]. In our study, for small-size pheochromocytoma (< 6 cm), postoperative hospital stay was similar between the two modalities. For large-size pheochromocytomas (≥ 6 cm), PRA could shorten the length of hospital stay, which may be because PRA did not enter the abdomen and did not interfere with the abdominal cavity. Gockel et al. compared these two surgical approaches for pheochromocytoma and showed similar perioperative outcomes between PRA and LTA, but this study lacked classification of tumor size [14]. Similarly, our study compared LTA with PRA in treating patients with small-size chromaffin cells (< 6 cm). It showed that both LTA and PRA were safe and effective with comparable surgical and postoperative outcomes. In a separate prospective investigation involving 77 patients with adrenal tumors subjected to PRA and LTA, the findings leaned in favor of recommending LTA for appropriate adrenal neoplasms [19]. Some scholars posit that PRA circumvents abdominal irritation and curtails the risk of bowel injury. Nevertheless, the retroperitoneal route may constrain surgical space, particularly when addressing larger and more intricate pheochromocytomas [20].

The realm of laparoscopic surgery brings forth an array of benefits, encompassing diminished complications, alleviated postoperative discomfort, and hastened recovery. While this minimally invasive approach has emerged as the preferred course for addressing petite pheochromocytomas, its endorsement for dimensions surpassing 6 cm raises reservations [13]. Therefore, LA for bulky pheochromocytoma remains controversial. Although LA is safe and effective for bulky pheochromocytoma [21, 22], it is unclear which laparoscopic approach is superior for bulky pheochromocytoma, and no relevant studies are comparing LTA with PRA for bulky pheochromocytoma, which may be due to the low incidence of pheochromocytoma and the difficulty in collecting bulky pheochromocytoma. In the context of this study, a direct comparison was made between PRA and LTA methodologies among patients harboring substantial pheochromocytomas (≥ 6 cm). The findings distinctly underscored PRA’s ascendancy over LTA in aspects spanning operation duration, intraoperative blood loss, postoperative complications, and the financial dimensions of postoperative hospitalization. Furthermore, it was revealed that LTA functioned as an independent factor in influencing operative durations surpassing the 150-min mark. It is imperative, however, to acknowledge that this does not preclude the likelihood that a surgeon’s proficiency in PRA could exert an impact on procedural outcomes.

The riskiest stage in pheochromocytoma surgery is between the beginning of contact with the tumor for dissection to complete tumor resection. Simultaneously, the circulatory system is the least stable before central adrenal vein transaction and requires stable blood pressure and expanded blood volume at any time [23, 24]. Some researchers have proposed that laparoscopic pheochromocytoma resection has more advantages than open surgery. However, intraoperative hemodynamics is a worrisome problem [25]. Thus, during this intricate maneuver, surgical expediency is pivotal to forestall undue tumor compression and traction. The PRA approach affords the surgeon-accelerated dissection along the anatomically avascular zone. This locale is readily discernible, attenuates tumor stimulation, facilitates blood pressure and heart rate control, and enhances hemodynamic equilibrium. Yu, Han, Zhou, Liu, and Ding have advocated for the prophylactic administration of phentolamine, revealing its efficacy in maintaining intraoperative hemodynamic stability [26]. Our outcomes underscore the inevitable fluctuations in intraoperative hemodynamics during laparoscopic pheochromocytoma surgery, necessitating the adept management of experienced anesthesiologists to ensure stability. Employing the scalpel for hemostasis in instances of tumor vessel rupture and bleeding during surgery stands as a viable strategy [27]. In case of emergency, the disposable absorbable clip can be used for clamping decisively.

Our study encompassed the proficient execution of laparoscopic procedures for 118 patients, even in instances where tumor dimensions reached 12 cm. Yet, it is imperative to acknowledge the study’s limitations, given its non-prospective nature and the absence of randomized comparison between surgical groups. To substantiate our findings, an extensive array of randomized controlled trials is warranted. Furthermore, despite the inclusion of 118 pheochromocytoma cases, the modest sample size within each subgroup demands vigilance against potential bias during subgroup analysis. Lastly, we acknowledge the regrettable absence of long-term follow-up in our study, a facet that would enriched the prognostic insights derived from our investigation.

## Conclusion

In summation, this investigation underscores the safety and efficacy of LTA and PRA in the hands of proficient laparoscopic practitioners, yielding comparable operative outcomes for patients harboring pheochromocytomas of size < 6 cm. In instances where tumor dimensions reached or exceeded 6 cm, both LTA and PRA demonstrated safety profiles. However, PRA emerged as the front-runner, distinguished by its abbreviated operation time, reduced intraoperative hemorrhage, diminished incidence of postoperative complications, and lower hospitalization expenses in contrast to LTA. Consequently, in the domain of patients afflicted with pheochromocytomas of size ≥ 6 cm, PRA emerges as the more advantageous approach.

## Data Availability

All data are available from the corresponding author.
